# Application of an Index Based on Aquatic Insect Biotic Integrity for River Ecosystem Health Assessment in Haihe River Basin, China

**DOI:** 10.3390/biology15171529

**Published:** 2026-09-03

**Authors:** Fanqing Kong, Zhilin Li, Yue Shen, Yanchu Zhao, Yin Hou, Zihang Hu, Shaowei Bian

**Affiliations:** 1 Ecological Environment Monitoring and Scientific Research Center of Haihe River Basin and Beihai Sea Area, Ministry of Ecology and Environment, Tianjin 300170, China; fanqingkong@163.com (F.K.);; 2 Zhang River, Wei River and South Canal Administration Bureau, Dezhou 253040, China

**Keywords:** aquatic insects organism, index of biotic integrity, aquatic ecosystem monitoring and assessment, river ecosystem, bioindicators

## Abstract

As critical biological components of freshwater ecosystems, aquatic insects are highly sensitive to cumulative anthropogenic disturbances and can effectively reflect long-term variations in river ecological quality, serving as ideal bioindicators for aquatic ecological monitoring and assessment. However, most existing benthic biotic integrity indices integrate diverse macroinvertebrate groups, which dilute the unique indicative value of aquatic insect communities, and few targeted evaluation systems have been independently developed for highly disturbed plain river basins in northern China. In this study, a field investigation of aquatic insect communities was conducted across 32 sampling sites in the Haihe River Basin from April to September 2023, aiming to construct a novel Aquatic Insect-based Index of Biotic Integrity (Ai-IBI) and systematically evaluate the basin’s river ecosystem health status. A total of 10,267 aquatic insect individuals belonging to eight orders and 43 families were identified, with Chironomidae dominating the community and predominantly distributing in midstream and downstream reaches. Based on ecological principles, discriminant ability analysis, and redundancy analysis, four core metrics were ultimately screened out to establish the Ai-IBI system, including total taxa richness, percentage of the top three dominant taxa, density of tolerant groups, and percentage of predators. Considering the widespread background ecological degradation of the Haihe River Basin, a localized reference-site selection framework integrating water quality standards, physical habitat assessment, and field ecological surveys was adopted to determine eight minimally disturbed reference sites, which effectively improved the regional applicability of the evaluation model. The health assessment results indicated that only a small proportion of river reaches maintained a healthy state, while most sites were classified as sub-healthy or fair, presenting a distinct spatial differentiation pattern wherein upstream mountainous reaches exhibited superior ecological integrity compared with downstream plain reaches. The constructed Ai-IBI showed high consistency with conventional physicochemical indicators and habitat evaluation results, verifying its excellent reliability and robustness. Different from traditional empirical IBI models suitable for near-natural watersheds, the Ai-IBI fully adapts to the unique ecological background of long-term water resource overexploitation, urbanization-induced habitat fragmentation, and nutrient enrichment in the Haihe River Basin. The Ai-IBI developed in this study provides a refined biological evaluation tool for quantitative diagnosis of river ecological degradation, offers empirical support for differentiated ecological protection and precise restoration strategies in the Haihe River Basin, and serves as a feasible methodological reference for ecological health assessment in other human-dominated plain river basins globally.

## Simple Summary

Rivers supply water for drinking, farming and industry, yet urban growth, water overuse, and pollution are harming the creatures that live in them. Small aquatic insects, such as the larvae of mayflies, dragonflies, and midges, spend part of their lives in riverbeds and are highly sensitive to environmental change, making them excellent early-warning signs of river health. However, most existing monitoring tools lump many different bottom-dwelling animals together, which masks the unique information insects can reveal. In this study, we collected more than 10,000 aquatic insects from 32 sites across the Haihe River Basin in northern China and built a new health index based on aquatic insects. The index combines four simple measures: the number of insect types present, how strongly a few common types dominate, the abundance of pollution-tolerant groups, and the share of predatory insects. Our assessment showed that only a small proportion of river reaches are healthy, while most are moderately degraded, with upstream mountain rivers in better condition than downstream plain rivers. This insect-based index gives managers a reliable way to detect river degradation early and guide protection and restoration, and can be adapted to other heavily used river basins around the world.

## 1. Introduction

Freshwater ecosystems worldwide are subjected to a complex array of anthropogenic stressors that operate across multiple spatial and temporal scales. Urbanization, for instance, drastically alters catchment hydrology through the expansion of impervious surfaces, increases pollutant loadings, and degrades instream habitats, collectively leading to profound changes in aquatic community structure [1,2]. Moreover, urbanization, flow regulation and water abstraction, industrial pollution, and land-use change each exert impacts of varying magnitude on river ecosystem health [3,4,5,6,7]. River ecosystem health assessment methodologies have evolved considerably, ranging from physicochemical measurements to comprehensive multi-dimensional integrity evaluations. Physicochemical monitoring relies on conventional water quality parameters to provide a rapid snapshot of ambient conditions at the time of sampling; however, its primary limitation lies in its inability to capture episodic pollution events or cumulative ecological changes [8,9]. Physical habitat assessment complements this by quantifying structural attributes of the riverine environment, including substrate composition, channel morphology, riparian vegetation cover, and flow characteristics, thereby reflecting habitat suitability and physical integrity [10]. As a crucial supplement, biological monitoring employs aquatic organisms as integrative indicators of environmental stress, offering a perspective on ecological conditions integrated over extended periods, thus overcoming the temporal constraints of instantaneous physicochemical data [11,12].

Freshwater systems are rich in biological diversity, and aquatic insects are important freshwater organisms who spend the immature larval stage of their life in water. Common aquatic insects, such as dragonflies, chironomids and mayflies, play a significant role in the processing and cycling of nutrients. Aquatic insects exhibit diverse feeding group traits, including predators, filter feeders, and collector gatherers. Bio-monitoring has several advantages because physicochemical data reflect the condition of the ecosystem only during the time of sampling, while bio-monitoring gives an indication of the long-term condition of the ecosystem due to the sedentary nature of benthic organisms [13].

Modern river ecosystem health assessment predominantly relies on multimetric biotic integrity frameworks, which effectively reflect long-term cumulative anthropogenic disturbances including hydrological alteration, urbanization, and nutrient enrichment [14,15,16]. Aquatic insect assemblages are regarded as core bioindicators for diagnosing subtle ecological degradation and evaluating the functional integrity of lotic ecosystems in both European and North American watersheds [14]. Macroinvertebrate-based biotic indices are core tools for freshwater ecological health evaluation worldwide. Single-metric indicators, including Family-level Biotic Index (FBI) and Ephemeroptera, Plecoptera, and Trichoptera (EPT) richness rely on aquatic insect pollution tolerance to rapidly diagnose organic contamination, yet they ignore community functional traits and overall structural integrity [17,18]. Multimetric frameworks such as the BMWP scoring system and Benthic Index of Biotic Integrity (B-IBI) integrate richness, density and feeding group metrics to reflect cumulative anthropogenic disturbance, which have become mainstream standardized assessment protocols [19,20]. In particular, the IBI and similar indices have been extensively applied across North America, Europe, China, and other regions, and the types of organisms measured have also expanded from benthic macroinvertebrates to encompass phytoplankton, periphyton, etc. [19,21,22,23,24]. The Benthic Index of Biotic Integrity (B-IBI) has been widely adopted and applied in riverine ecosystems, where it has been useful in providing scientifically defensible evidence of environmental conditions [25,26] and developing biological criteria for stream protection and restoration [27,28].

Despite the proven utility of B-IBI in freshwater ecological condition assessment, reports on aquatic insects as indicators for the construction of an index of biotic integrity are rare. Some insect groups, such as EPT, are highly sensitive to water pollution, while some groups, such as Chironomidae, can survive and proliferate in extremely disturbed or polluted environments [29]. Aquatic insects, particularly those with specific flow requirements such as lotic-adapted Ephemeroptera, Plecoptera, and Trichoptera species, are sensitive indicators of hydrological alteration [30]. Flow reduction and increased flow variability have been shown to reduce taxonomic richness and shift community composition toward tolerant generalists [31,32]. They are vital indicators of the changes in freshwater habitats, and family-level identification can be valuable in evaluating the quality of ecological conditions [33].

Traditional multimetric biotic indices integrate diverse benthic groups and dilute the sensitive taxonomic, functional and pollution-indicative signals unique to aquatic insect assemblages. General benthic integrity tools cannot precisely capture the subtle gradients of anthropogenic disturbance reflected by EPT, chironomids, and functional feeding groups of aquatic insects. Therefore, it is urgently necessary to develop an independent integrity index specifically targeting aquatic insects, which enables refined diagnosis of freshwater degradation and provides targeted technical support for standardized water ecological health monitoring.

The Haihe River Basin is a typical water-scarce plain basin in northern China, suffering from intensive human interference, long-term water resource overexploitation, widespread habitat degradation, and basin-wide background pollution. In addition, the basin represents the most common degraded river type in northern China, making it highly suitable for constructing a targeted insect-based ecological integrity evaluation system that can be applied to similar basins. It represents the most common degraded river type in northern China and is highly suitable for constructing a targeted insect-based ecological integrity evaluation system. The objective of this study was to develop an aquatic insects based on aquatic insects for river ecosystem health assessment in the Haihe River Basin.

## 2. Materials and Methods

### 2.1. Study Area

The Haihe River Basin is one of the seven major river basins in China, situated in the eastern part of the country, covering a total area of 318,000 km^2^. As of 2020, the predominant land-use types in Haihe River Basin are cultivated land, forest, and grassland, constituting 45.60%, 21.30%, and 16.70% of the total area, respectively. Cultivated land is predominantly found in the eastern region of the basin, while forest and grassland are primarily distributed in the northwest and southeast regions. The Haihe River Basin has the largest water system in North China, and it plays an important role in contributing to economic and social development. The lower reaches of this area are located at the junction of the North China Plain and the Bohai Sea. There are many developed cities and strong human activities in this area, while the rapid development of cities has led to issues such as overexploitation of water resources, fragmentation of aquatic habitats, and eutrophication of water bodies, which are significant problems facing the aquatic ecosystems in the Haihe River Basin.

**Figure 1:**
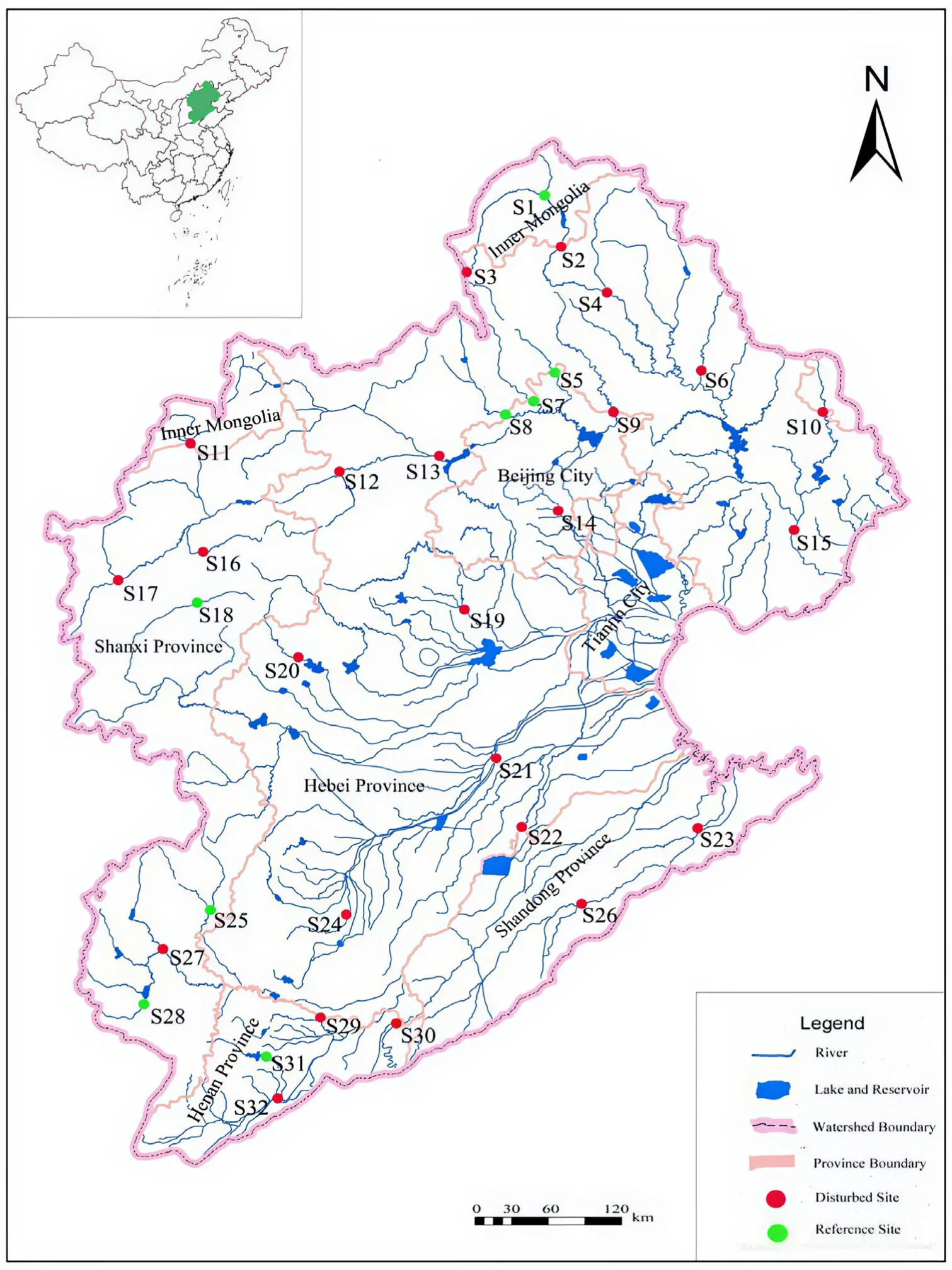
Map of Haihe River Basin and sampling sites.

From April to September 2023, we collected aquatic insect samples from 32 river sites distributed throughout the Haihe River Basin. The sampling sites set up in this study were carefully selected to ensure representativeness, covering rivers of all stream orders as well as both mountainous and plain areas across the basin, so as to comprehensively characterize the status of aquatic biodiversity within the basin. The sites were selected on the basis of accessibility as well as the need to include sites that represented both reference and disturbed conditions (refer to Figure 1).

### 2.2. Aquatic Insect Sampling

All field samplings were consistently conducted by two professional ecological investigators with unified sampling training and standardized operational procedures to ensure sampling consistency and reduce human-induced errors. The entire field sampling campaign lasted for 15 consecutive days during the investigation period. To eliminate diurnal variation interference on aquatic insect community collection and guarantee the comparability of sampling data, all field sampling activities were uniformly performed during the daytime period from 8:30 a.m. to 15:00 p.m. No nighttime sampling was conducted, and all samples were collected in the same daily time window, which effectively avoided community differences caused by the diel migration and activity rhythm of aquatic insects.

Aquatic insects were collected by using the “kick net method” (mesh opening: 425 μm; area 1 m^2^) and “suber net method” (mesh opening: 425 μm; area 0.09 m^2^) in wadable rivers and the “Peterson sampler method” (area 0.0625 m^2^) in non-wadable rivers [10]. To avoid duplicate sampling of the same microhabitat, we systematically divided the 100 m sampling reach into distinct microhabitat zones (riffles, pools, and vegetated margins) and allocated sampling effort proportionally to the availability of each microhabitat type within the reach. Within each microhabitat type, replicate samples were taken at least 5 m apart to ensure spatial independence. The three replicate samples per station were collected consecutively, with the sampling net thoroughly rinsed between replicates to prevent carryover of organisms. The triplicate samples were pooled to make one composite sample per site. Aquatic insect samples were stored in 100 mL plastic bottles containing 75% ethanol, labeled with the respective field data in order to be processed in the laboratory. Collected samples were examined under a microscope (Zeiss Imager M2) (Carl Zeiss Microscopy GmbH, Jena, Germany) and stereo microscope (Nikon SMZ25) (Nikon Optical Co., Ltd., Shinagawa, Japan). Aquatic insect specimens were separated into systematic groups, followed by available keys and standard taxonomic literature to the lowest possible level [34,35,36,37,38].

### 2.3. Determination of Reference Sites and Disturbed Sites

The reference sites that represent a group of minimally disturbed sites need to be established when developing a multimetric index. According to Chinese environmental quality standards for surface water, surface water quality can be separated into 6 grades from good to bad, that is I, II, III, IV, V and inferior V. The habitat was assessed by a scoring method based on 10 metrics, such as substrate, habitat complexity, velocity–depth combination, etc., and it can be scored from 0 to 200 [35]. Reference sites were chosen which satisfied the 4 conditions as follows: the water quality was better than grade II, integrated habitat assessment index scored greater than 120, human disturbance was minimal, and riparian land was not cultivated. Here, water quality grade was based on the Chinese environmental quality standards for surface water [39]. In this study, 8 sampling sites, Baichengzi (S1), Dacaoping (S5), Sidaodian (S7), Houcheng (S8), Xiaruyue (S18), Xiajiaozhang (S25), Situqiao (S28) and Huanghuaying (S31), were designated as reference sites. It is believed that these areas experience less human interference and possess better habitat conditions, and the other 24 sites were disturbed sites.

### 2.4. Metric Selection

**Figure 2:**
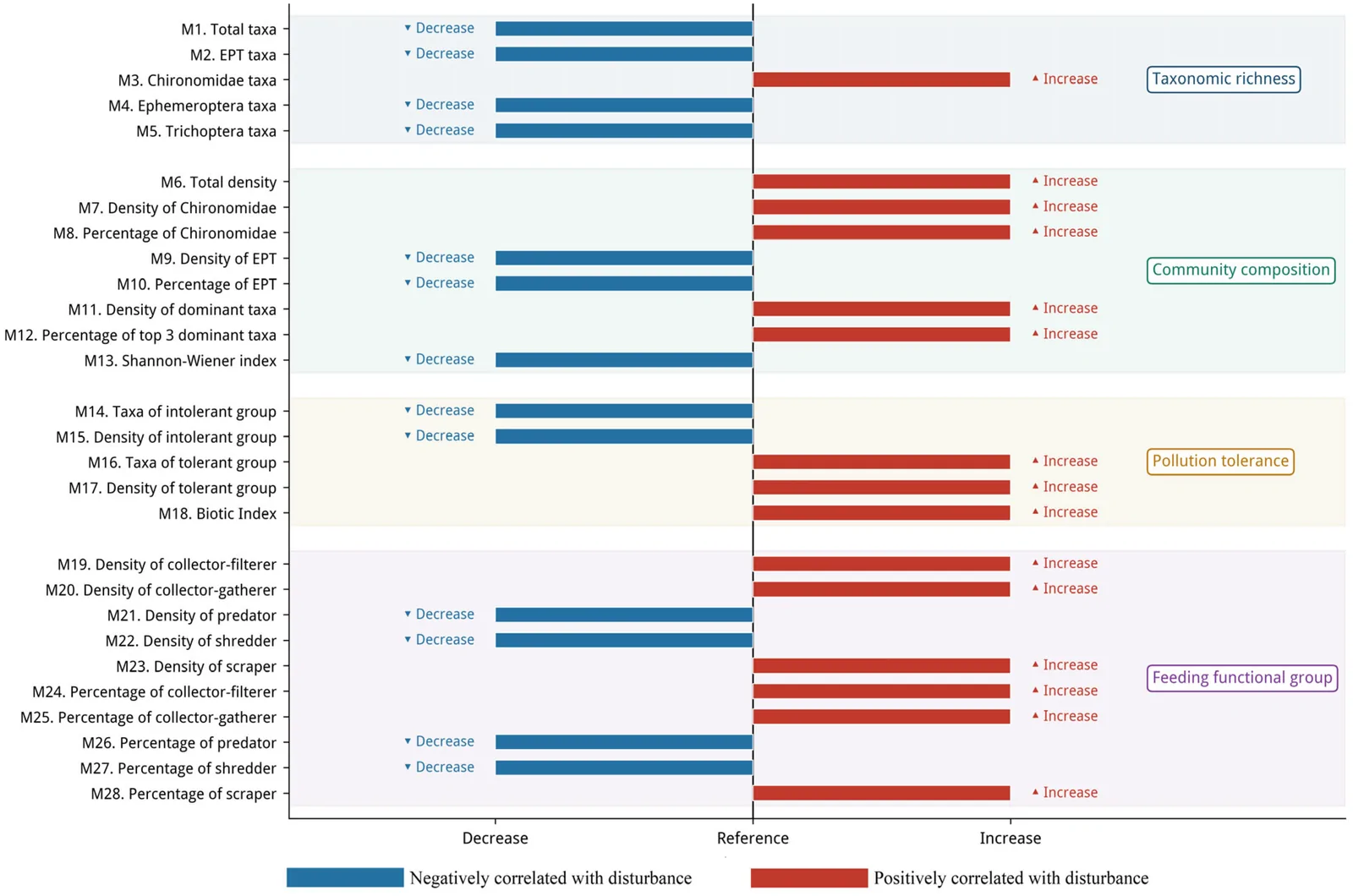
Candidate metrics and their expected response to disturbance.

Focusing on the actual situation faced by the aquatic ecosystem in the Haihe River Basin, and the principles for selecting candidate metrics (that they should (1) be based on well-understood ecological principles relevant to the biological community in the type of water body being studied, as well as sampling methods and assessment objectives; (2) respond to artificial stress in a predictable manner; (3) have responses to stressors that can be distinguished from natural variation and that can discriminate along a gradient of artificial stress; (4) are environmentally benign to measure; and (5) are cost-effective to sample), 26 candidate biological metrics in 4 categories were chosen, including taxonomic richness, community composition, pollution tolerance and feeding functional group. Expected responses to disturbance of candidate biological metrics were analyzed (refer to Figure 2), in which tolerance and intolerance measures were calculated by sorting families on the basis of the tolerance values of insects. Families with a tolerance value of 7.0 and above were categorized as tolerant organisms and intolerant taxa were taken as the sum of EPT. Percentage composition of filterers, grazers and scrapers were calculated as feeding metrics. Pollution tolerance of insects and the other measures used in this study have been adopted from the modified values for the Asian region [26].

### 2.5. Discriminant Ability and Redundancy Analysis

Discriminant ability analysis was conducted using the box plot method to compare the interquartile range (25% to 75% quartile box) and median overlap between reference and impacted sampling points, assigning different scores based on the overlap. When the interquartile range is ≥2, the metric is considered to have environmental pressure-discriminant ability and is used for further analysis. Redundancy analysis: Conduct a normality test on the metrics that meet the requirements. If these metrics all follow a normal distribution, use Pearson correlation analysis; if not normally distributed, use Spearman correlation analysis, retaining metrics with correlation coefficient |r| < 0.75.

### 2.6. Metric Scoring and Index Development

The values of the 4 metrics were transformed into a uniform score, and then calculated by ratio scoring method [19,21].

Metric scoring was based on a continuous scoring method [40,41]. Each metric’s raw value at each sampling site was individually scored on a continuous scale (0–100) reference gradient scoring approach. Likewise, the reference gradient scoring approach uses the 5th and 95th percentiles of each metric’s raw value in the reference community samples in a given region to create a linear scale. For metrics that decrease in value with disturbance, the 5th percentile of the reference was assigned a score of 0 and the 95th percentile of the reference was assigned a score of 100. Metric values less than or equal to the 5th percentile and greater than or equal to the 95th percentile were scored 0 and 100, respectively. Metric values between the 5th percentile and 95th percentile were scored proportionally from 0 to 100 according to the following equation:

$$Score=\;\frac{X-X95re}{X95ref-X5ref}$$

re = recorded value; ref = reference value.

*X* is the observed value of the metric at each sampling station, *X*95 is the 95th percentile value, and *X*5 is the 5th percentile value. The reverse was done for metrics that increase with disturbance. Metric values less than or equal to the 5th percentile of the reference are given a score of 100, values greater than or equal to the 95th percentile of the reference are scored 0, and values between the 5th percentile and 95th percentiles are scored proportionally according to the equation

$$Score=\frac{X95ref-X}{X95ref-X5ref}$$

ref = reference value.

## 3. Results

### 3.1. Aquatic Insect Community Structures

**Figure 3:**
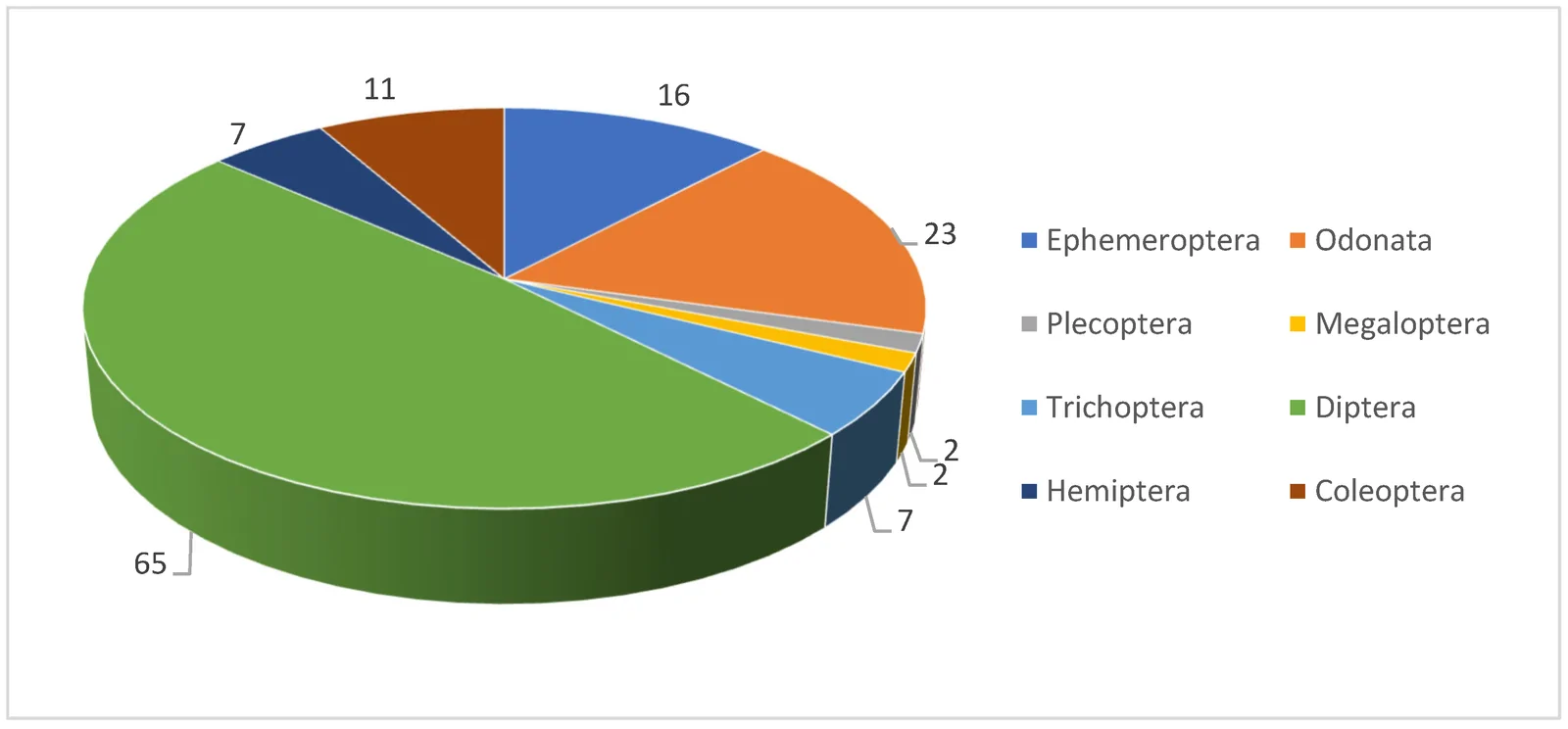
Species number of the 8 orders of the aquatic insects from the rivers of Haihe River Basin.

A total of 10,267 individual aquatic insects belonging to 133 species, 43 families, and eight orders (Ephemeroptera, Odonata, Plecoptera, Trichoptera, Megaloptera, Diptera, Hemiptera and Coleoptera) were collected from the rivers in the Haihe River Basin (refer to Figure 3). Chironomidae belonging to Diptera contributed more than 50% of the total aquatic insect abundance and mainly occurred at the midstream and downstream sites. Taxa richness ranged from 3 to 23. The reference site was rich in taxa (18 on average), while the most disturbed site was represented by only 3 taxa. As a representative indicator of clean water, the number of EPT taxa showed low biological diversity which ranged from 0 to 8, and the plain areas usually monitored low number of EPT species. The result showed significant density differences among the sites in aquatic insects from 23 ind./m^2^ to 1693 ind./m^2^. The Shannon–Wiener diversity index varied from 0.77 to 3.62.

### 3.2. Determination Process of Core Metrics

**Figure 4:**
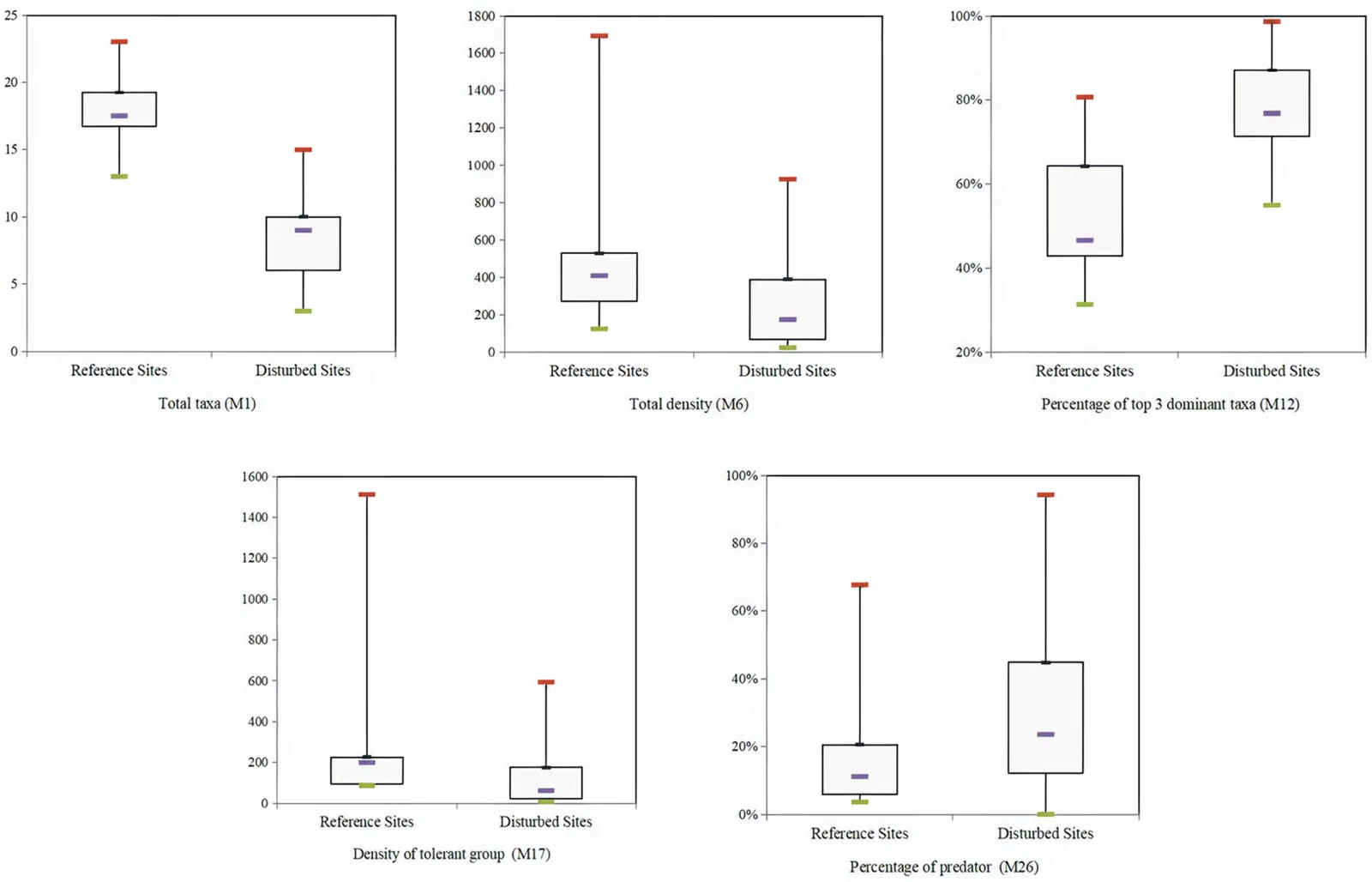
Box plots of 5 candidate metrics between reference and disturbed sites.

Discriminant ability analysis was conducted using box plots to compare the interquartile ranges (25% to 75% quartile box) and median overlaps between the reference sites and disturbed sites. The results indicate that metrics M1, M6, M12, M17 and M26 showed an interquartile range IQ ≥ 2 in their box plots (refer to Figure 4), and hence, they were retained. All other metrics were excluded.

Redundancy analysis indicates that due to the strong correlation between metrics M1 and M6, and the fact that M1 reflects the stability of benthic animal community structure and carries more information, M1 was retained. Consequently, four metrics were selected for constructing the Ai-IBI index for the rivers of Haihe River Basin: total taxa (M1), percentage of top three dominant taxa (M12), density of tolerant groups (M17) and percentage of predators (M26).

### 3.3. Spatial Distribution of River Ecosystem Health Based on Ai-IBI

The Ai-IBI value was obtained by summing up all the four metrics. Formulas for calculating the four metric scores were shown as Table 1, and the criteria of aquatic insects-index of biotic integrity (healthy state) was shown as Table 2.

The health assessment results of the Haihe River Basin using the Ai-IBI indicated that 4 sites were in a healthy state; 10 were sub-healthy; 11 were fair; 5 were poor; and 2 sites were in a very poor state (refer to Table 3). The river ecosystem health assessment results based on the Ai-IBI revealed that the upstream sites and river reaches presented better ecological conditions than the downstream ones, and the reference sites exhibited significantly higher biotic integrity than the disturbed sites. This spatial pattern can be primarily attributed to the superior overall aquatic ecological environment quality in the upper reaches of the Haihe River Basin. Compared with the downstream areas, the upper reaches experience less intensive urbanization and suffer from lower anthropogenic disturbance on aquatic ecosystems.

## 4. Discussion

The concept of the multimetric index of biotic integrity (IBI), originally formulated for fish assemblages in midwestern United States streams [21], has been progressively extended to benthic macroinvertebrates and extensively adapted across continents, giving rise to a diverse array of region-specific assessment tools. In North America, the Benthic IBI (B-IBI) developed for rivers of the Tennessee Valley [19] established a widely adopted framework integrating taxonomic richness, compositional attributes, trophic structure, and pollution tolerance, which was subsequently refined for diverse ecoregions including Florida streams [39] and Pacific Northwest streams [13]. In Europe, implementation of the Water Framework Directive has stimulated a remarkable proliferation of biological assessment methods, with 297 methods reported across 28 countries, more than half of which rely on benthic invertebrates and aquatic plants [42]; among these, standardized multimetric indices developed through the AQEM and STAR projects [43] constitute the mainstream approach for assessing river ecological status. In South Africa and the broader Afrotropics, rapid bioassessment tools such as the South African Scoring System version 5 (SASS5), which assigns family-level sensitivity scores to infer water quality [44], remain the dominant practice, while macroinvertebrate-based IBIs have also been developed and validated for tropical rivers [45]. The Aquatic Insect-based Index of Biotic Integrity (Ai-IBI) developed in this study enables holistic, dynamic, and long-term tracking of river ecosystem health, particularly suitable for the highly disturbed and anthropogenically modified Haihe River Basin [46,47]. Traditional water quality assessments often fail to reflect latent ecological degradation caused by chronic low-intensity disturbances, whereas biotic indicators can effectively identify early ecosystem declines that are undetectable by routine water quality monitoring, thereby compensating for the limitations of physicochemical evaluation systems.

In this study, the Ai-IBI was established through data-driven indicator screening and quantitative threshold optimization, which effectively avoids the subjective artificial classification and empirical threshold setting commonly adopted in conventional IBI construction procedures. This standardized and quantitative modeling framework significantly improves the numerical accuracy, spatial comparability, and repeatability of river health evaluation results. More importantly, compared with single physicochemical indicators that merely reflect instantaneous water environment conditions, community-based biotic indices can comprehensively integrate the combined ecological effects of water pollution, habitat fragmentation, hydrological regime changes, and land-use disturbance. The Ai-IBI therefore provides a more systematic and ecologically meaningful evaluation of basin-scale ecosystem degradation, which is critical for accurately characterizing the overall ecological status of severely impaired river basins.

The selection of representative reference sites is widely recognized as the core and prerequisite step for reliable B-IBI development, as reference conditions provide the baseline benchmark for distinguishing ecological differences between minimally disturbed and impaired river reaches. Ideally, reference sites should maintain near-natural ecological conditions with negligible human interference to reflect the inherent ecological characteristics of regional river ecosystems. However, highly urbanized and densely populated watersheds such as the Haihe River Basin face inevitable challenges in defining absolute reference conditions. Long-term intensive industrial development, agricultural fertilization, urban expansion, and excessive water resource exploitation have resulted in widespread background ecological impairment across the entire basin, with almost no completely undisturbed river reaches remaining. This phenomenon makes the traditional strict reference-site selection criteria inapplicable for highly developed plain river basins.

To address this regional dilemma, the present study adopted a comprehensive and localized reference-site screening framework that integrated actual field ecological surveys, national surface water environmental quality standards, and systematic river habitat integrity assessments. Through multi-dimensional quantitative evaluation of water quality, physical habitat structure, and aquatic community composition, eight representative sites with relatively minimal anthropogenic disturbance and relatively complete ecological functions were finally determined as the basin-specific reference conditions. This optimized selection strategy abandons the rigid natural-basin reference standard and establishes disturbance adaptive reference benchmarks suitable for the degraded Haihe River Basin, which greatly enhances the rationality and regional applicability of the evaluation system. However, given the widespread ecological degradation across the Haihe River Basin, the reference sites established in this study represent “least-disturbed” rather than “pristine” conditions, which may compromise the sensitivity of the index at the upper end of the ecological health gradient. In addition, the Ai-IBI does not explicitly incorporate flow regime metrics, despite discharge being a critical driving factor for aquatic insect communities; future refinements could integrate hydrological indicators to further enhance the predictive capacity of the index.

The Ai-IBI constructed in this study demonstrates prominent rationality, robustness, and practical applicability for ecological health assessment in human-dominated river basins. Different from conventional IBI systems that are mostly established for natural or slightly disturbed watersheds and lack adaptability to heavily degraded aquatic environments, the Ai-IBI fully considers the unique regional ecological background of the Haihe River Basin, including long-term water resource overexploitation, severe habitat degradation caused by urbanization, widespread nutrient enrichment, and cumulative basin-wide ecological stress. In the indicator screening process, the initial candidate metrics were strictly filtered based on their environmental sensitivity, spatial discriminatory power, and community stability under regional disturbance gradients. Redundant indicators and low-sensitivity metrics that cannot effectively reflect basin ecological degradation were eliminated, ensuring that the final evaluation system can accurately capture the spatial differentiation characteristics of ecological health in the Haihe River Basin.

Furthermore, the high consistency between Ai-IBI evaluation results and traditional physicochemical indices, including organic pollution parameters and eutrophication indicators, as well as field investigations of aquatic community and physical habitat quality [48,49], further verifies the reliability and validity of the newly constructed index. The complementary relationship between biotic evaluation and physicochemical evaluation indicates that the Ai-IBI can not only reflect surface water pollution status but also characterize potential ecological degradation caused by long-term habitat damage. Overall, this study provides a localized, robust, and adaptable biotic integrity evaluation tool for ecological assessment and restoration management of the Haihe River Basin, and it offers a feasible methodological reference for health evaluation in other highly disturbed plain river basins worldwide.

## 5. Conclusions

This study systematically evaluated the aquatic ecological health of the Haihe River Basin using aquatic insects integrity index. The results demonstrated significant spatial heterogeneity in basin ecological quality. Specifically, the upstream reaches presented remarkably better ecological health conditions than the downstream reaches, reflecting a consistent spatial pattern whereby mountainous areas sustain superior aquatic ecosystem status compared with plain areas. Such spatial differentiation is predominantly driven by the combined effects of natural geographic variability and gradient differences in anthropogenic disturbance intensity across the basin.

The better ecological integrity observed in mountainous upstream regions is primarily attributable to well-preserved hydrological regimes and riparian habitats. Upstream river segments maintain natural flow variability, diverse substrate conditions, and intact riparian vegetation, which provide stable habitats, feeding grounds, and breeding environments for aquatic insects. These favorable habitat characteristics support high biodiversity, abundant aquatic insect assemblages, and stable community structures, thereby sustaining high-level aquatic ecological integrity. Furthermore, mountainous upstream areas experience relatively low human disturbance, with limited land-use transformation, weak industrial pollution, and moderate water resource exploitation, which minimizes exogenous interference with benthic communities.

In contrast, downstream plain regions are subject to intensive and long-term anthropogenic pressures, resulting in substantial aquatic ecological degradation. Dense urbanization, intensive agricultural irrigation, and concentrated industrial activities in plain areas have induced a series of ecological problems, including reduced river runoff, widespread habitat fragmentation, water eutrophication, and continuous pollutant accumulation. These environmental alterations substantially disrupt the living conditions of aquatic insect communities, leading to the decline of sensitive indicator species, simplification of community composition, and decreased ecosystem stability, which collectively degrade the overall water ecological health of downstream basins.

This study clarifies the spatial differentiation characteristics of aquatic insect-based ecological health in the Haihe River Basin, providing empirical support for the formulation of targeted watershed ecological protection and restoration strategies. For future watershed management, priority should be given to habitat conservation and natural hydrological process maintenance in mountainous upstream zones. In downstream plain areas, pollution remediation, habitat rehabilitation, and ecological flow supplementation should be emphasized to reverse ecological degradation and promote the sustainable improvement of aquatic ecosystem quality in the Haihe River Basin.

## Figures and Tables

**Table 1:** Formulas for calculating 4 metric scores by ratio scoring method.

Metrics	Equations for Scores	Source of Denominator Values
Total taxa (M1)	M1/15	15 = 95th percentile of reference distribution
Percentage of top 3 dominant taxa (M12)	(98.57%–M12)/(98.57%–31.38%)	98.57% = 95th percentile; 31.38% = 5th percentile of reference distribution
Density of tolerant group (M17)	(1510.97–M17)/(1510.97–6.67)	1510.97 = 95th percentile; 6.67 = 5th percentile of reference distribution
Percentage of predator (M26)	M26/0.43	0.43 = 95th percentile of reference distribution

**Table 2:** Criteria used to evaluate ecosystem health.

Value of Ai-IBI	Ecosystem Health Status
>2.96	Healthy
2.22–2.96	Sub-healthy
1.49–2.22	Fair
0.75–1.48	Poor
<0.74	Very poor

**Table 3:** Ai-IBI scores and healthy states of 32 sites in the Haihe River Basin.

No.	Sites	Reference/Disturbed Sites	Score	Healthy State
S1	Baichengzi	RS	2.47	Sub-healthy
S2	Dahekou	DS	2.77	Sub-healthy
S3	Shandianhe Bridge	DS	3.09	Healthy
S4	Guojiatun	DS	1.98	Fair
S5	Dacaoping County	RS	2.97	Healthy
S6	Shangerdaohezi	DS	2.04	Fair
S7	Sidaodian	RS	3.11	Sub-healthy
S8	Houcheng	RS	3.25	Healthy
S9	Gubeikou County	DS	2.39	Sub-healthy
S10	Hutou Stone	DS	0.72	Very poor
S11	Puziwan	DS	2.41	Sub-healthy
S12	Huliu River	DS	2.65	Sub-healthy
S13	Number Eight Bridge	DS	1.32	Poor
S14	Shawo	DS	1.89	Fair
S15	Luanxian Bridge	DS	2.25	Sub-healthy
S16	Si County	DS	2.11	Fair
S17	Mayi	DS	2.69	Sub-healthy
S18	Xiaruyue	RS	1.98	Fair
S19	Beihedian	DS	2.15	Fair
S20	Wanglinkou	DS	2.04	Fair
S21	Tiancun	DS	2.10	Fair
S22	Disandian	DS	2.13	Fair
S23	Fuguo County	DS	0.59	Very poor
S24	Shayang	DS	1.33	Poor
S25	Xiajiaozhang	RS	2.76	Sub-healthy
S26	Xiakou	DS	1.40	Poor
S27	Xiaojiao	DS	2.33	Sub-healthy
S28	Situ Bridge	RS	2.04	Fair
S29	Fengsu Bridge	DS	1.29	Poor
S30	Nanle Hydrostation	DS	1.60	Fair
S31	Huanghuaying	RS	3.37	Healthy
S32	Xiamaying	DS	0.98	Poor

RS: reference sites; DS: disturbed sites.

## Data Availability

The data that support the findings will be available in Zenodo (Invenio RDM v13.0) at 10.5281/zenodo.21530889 following an embargo from the date of publication to allow for commercialization of research findings.
